# Ameliorated New Media Literacy Model Based on an Esthetic Model: The Ability of a College Student Audience to Enter the Field of Digital Art

**DOI:** 10.3389/fpsyg.2022.943955

**Published:** 2022-08-04

**Authors:** Rui Xu, Chen Wang, Yen Hsu

**Affiliations:** ^1^The Graduate Institute of Design Science, Tatung University, Taipei, Taiwan; ^2^School of Art and Design, Fuzhou University of International Studies and Trade, Fuzhou, China; ^3^College of Journalism and Communications, Shih Hsin University, Taipei, Taiwan

**Keywords:** esthetic model, esthetic experience, new media literacy, digital art, college student audience

## Abstract

In the current digital environment, people can visit every corner of the world without leaving their homes. New media technology compresses distance and time, but it also subverts the traditional mode of audience presence. Many traditional, offline content expression modes are also moving toward the digital field, and digital art is among them. Digital new media is a new art form that requires its audience to have a new media literacy; this literacy is necessary for esthetic experience and for audience participation. At present, the relatively lack of objective methodology for scientific research on aesthetic and media literacy has limited our current understanding. Therefore, we need to develop a new model and conduct empirical research with college students as the audience. Empirical research was conducted with an audience of college students. The study had the following purposes: (1) to add a new dimension to the esthetic model, namely new media literacy, to align the model with the current digital environment, and (2) to test the moderating effect of new media literacy on esthetic emotion as represented by interest and confusion. The experiment verified the study’s hypothesis that higher new media literacy was associated with higher esthetic interest and lower confusion. By contrast, has a substantial influence on the cognitive processes in humans, lower new media literacy was associated with lower esthetic interest and higher confusion. New media literacy is an essential quality for contemporary audiences. This knowledge may be useful for effective design. It provides a traditional and favorable learning environment and empirical reference for the subsequent improvement of digital aesthetics and media literacy.

## Introduction

### Research Background

Distance education means that individuals can learn without leaving their homes. They can participate in virtual activities to accomplish feats that cannot be accomplished in real life. New media technology has markedly changed audience dynamics. The number of Chinese mainland mobile phone users reached 1011 million in December 2021 (Forty-eitghth China Internet Development Statistics Report 2021). With continuous improvement of digital technology and continuous expansion of the scale of Internet users, all types of information on new media are uneven. Concurrently, all fields of society are gradually advancing in the digital era. The pace of technological innovation is accelerating, and the supply of digital applications and resources is growing exponentially (Segers and Bauwens, 2010), providing opportunities for new digital media. Many traditional offline activities are also transferable to the digital field, and digital art is one of them.

The purpose of this study was to explore the esthetic experience of an audience entering the digital field. Last year, the 2021 Asia Digital Art Exhibition was officially opened at Beijing Times Art Museum. This art exhibition brought together many digital art works of international art masters and young generation Z, presenting a future world of oriental civilization and Asian culture, leading the audience to experience The operating space between fiction and the future, into the new world and new civilization generated by digital media. It means that more and more modern art begins to express with digital art and X Art Museum in the summer of 2020 was on of the rapid developing digital art field. The project was positioned as a super contemporary arts museum, focusing on new forces of Chinese and foreign art in a global context and integrating the characteristics of technology and art in digital art. The X Virtual Art Museum project of the art museum was displayed on its new media website. It explored the possibilities of a virtual art museum and modes of connection between online space and offline exhibitions (in addition to virtual reality [VR], which is a method of simulating and restoring reality). On the one hand, the project presents the artist’s attention and research on topics such as artificial intelligence, virtual reality, Internet images, and popular culture; on the other hand, the project also reflects the far-reaching impact of new media and cutting-edge technologies on people’s real-life relationship and social structure. The digital art works selected for this study were the first Triennial Exhibition “Terminal > _ How Do We Begin?,” which was planned by Wu Dongxue, the chief curator of X Art Museum. The museum has exhibited over 70 works by 33 artists, which have been displayed using digital art and technology or other interdisciplinary approaches. The audience must have certain skills to absorb media when they view an exhibition. They must click on, enlarge, shrink, and move the works and information they want to view to fully understand these works.

Digital art is a young, diverse, and rapidly developing art field. X Art Museum is only representative. Elsewhere, digital art has more novel expressions using unified digital tools, technical language, flexible digital communication carriers, and unlimited replication. In terms of time and space, digital art is far greater than traditional material media because it can lock the creator’s works and the audience’s attention by using the Internet as a medium. Furthermore, accessing digital media and making use of new network media is essential for individuals to enter the digital field. As a new art form, digital new media art embodies the characteristics of our times through the combination of art and science and people’s demands for the development of contemporary art. To enter the field of digital art, an individual must have literacy in operating and understanding new media. This is a new media literacy for audiences participating in digital art.

A new concept, new media literacy in the context of digital art involves helping people better understand media ecology in the digital environment (Lin et al., 2013). New media literacy is a set of skills that are regarded as “the ability to acquire, analyze, evaluate and disseminate information in various forms,” and such skills enable an audience to access and create various forms of information and to recognize the nature, importance, and basic value of information (Ghasemi et al., 2006). New media literacy can also be used to analyze and reflect on the esthetic components, institutional structure, economic background, and interactiveness of mass media (Silverblatt et al., 2015).

Continuous progress in digital technology may create new art forms, but it will not directly bring new art content. To experience digital new media art, we must use digital media to facilitate esthetic communication between audiences. With the development of digital media, indigo closely tracks the latest digital technology and obtains a new structure in the digital communication environment (Zhongli et al., 2007). In the digital environment, only with a certain new media literacy can we feel the uniqueness of art itself, helping works give their audience a new esthetic experience.

### Research Objective

People are highly immersed in the latest developments in the media environment. Coupled with the popularity of tablet computers and smartphones, new media has penetrated most aspects of daily life. In addition, with the rapid development of new media technology, reexamining the esthetic experience of digital art audiences is necessary.

Generally, new media literacy is widely considered to encompass a combination of information skills, traditional computer literacy, and communication skills (or multiple literacy); this combination requires media users to change from audiences, who passively accept information, to users, who actively consume information (Liu, 2017). This matches the literacy requirements for an audience to master media access skills and actively participate in digital art viewing.

Studies that have investigated esthetic experience have generally focused on the field of psychology, such as the esthetic interest and confusion discussed in Section “Interest and Perplexity as Esthetic Experience,” while ignoring the literacy requirements of an audience entering the esthetic field. Therefore, grounded in an esthetic experience combined with new media literacy theory, this study explored the esthetic experience model of an audience in the digital environment. The following were the research objectives:

On the basis of a literature review and research background, the study aimed to add a new dimension to the esthetic experience model (Silvia et al., 2013; Avalos et al., 2021) of new media literacy that is suitable for representing esthetic experiences in the digital environment.

The mediating effect of the proposed concept, new media literacy, in “esthetic emotion represented by interest and confusion” was tested.

## Literature Discussion

### Digital Art

Digital art is a comprehensive concept covering works of art and the creation process. In a broad sense, digital art is art through digital processing. Examples are graphic design by means of digital technology and mobile phone ringtones; as long as digital technology is used as the carrier and it has independent esthetic value, a work can be considered digital art. Currently, scholars interpret digital art from different perspectives. For example, it is considered “a new art form based on digital technology and modern media technology, which integrates people’s rational thinking and art’s perceptual thinking.” Some scholars define digital art as “making use of digital and information technology, with independent esthetic value, and ultimately still presented in the form of works of art.” Another view holds that “digital media art is a new direction for information science to expand into the field of culture and art, which is dominated by technology and supplemented by art, and is a new discipline combining technology and art” (Li, 2006).

In the process of creation, all (or part of) of a digital artwork is created using digital technology such as interactive media design, digital imagery, virtual reality, or new media. The authors believe that it is too early to define an art form as new as digital art in terms of elements such as its basic concept, existing state, essential characteristics, development law, creation method, artistic value, and communication and consumption mode.

In short, researchers have emphasized the new mode of art presentation afforded by new media technology. Therefore, the concept of digital art discussed in this study refers to works of art made using digital technology and for which new media platforms act as the exhibition space.

### Interest and Perplexity as Esthetic Experience

With the increased interest in emotional expression in art works (Armstrong and Detweiler-Bedell, 2008; Cupchik et al., 2009; Silvia, 2010), emotion has gradually become the most expressed aspect of art (Silvia, 2005a). These emotions are expressed through creation and shared through perception. In the history of art, the complex emotions of shock, sublimity, epiphany, and transformation caused by an encounter with art are collectively referred to as the esthetic experience. The esthetic experience involves positive and negative emotional states such as arousal, happiness, infatuation, admiration, and sadness. Pleasant and unpleasant emotions can exist simultaneously (Scherer and Wallbott, 2005). Moreover, such emotions are not limited to the pleasure of formal esthetics. When an esthetic experience is strong, it may trigger physiological reactions such as tears or goosebumps. With the gradually clearer division between daily and esthetic experiences, the psychological theory of the esthetic experience of art has further developed. Generally, changes in esthetic experience may be related to numerous variables. On the basis of the psychological theory of art appreciation, it is necessary to consider a function that may mediate the appreciation of works of art and other stimuli, namely the relationship between emotion and profession (Leder, 2003).

An evaluable emotion, interest can stimulate learning and exploration. If an artwork stimulates people to learn for their own interests, it can help them gain diverse knowledge, skills, and experience (Avalos et al., 2021). Interest evaluation methods involve two aspects, namely the evaluation of novelty and the complexity of stimuli. The novelty–complexity variable is in the first order of a multilevel sequential evaluation model. Its key concept is that the assessment event is new, unexpected, and unfamiliar. Numerous studies have shown that novelty–complexity variables have a substantial impact on interest, and, in many experiments, these variables have had an inverted-U shape (Silvia, 2005a). Although novelty–complexity is a primary concern, it is not the only variable closely related to interest. The comprehensibility of stimuli can also predict interest (Ellsworth and Scherer, 2003). As the second evaluation factor, intelligibility, also known as coping potential, refers to comprehensibility in the face of strangeness, complexity, and ambiguity. In many art and psychological studies, comprehensibility has been proved to affect interest as a coherent factor (Silvia, 2005a). For example, people are more interested in something when they think it is novel, complex, and understandable. In short, interest is a type of emotion generated by confronting the unknown but knowable that can stimulate love for new and complex things. However, when people come to understand and master something, they seem to become less interested in it.

Since the pioneering work of Berlyne (1971), interest (in terms of the esthetic experience and in experiential esthetics) has been widely studied (Tsutsui and Ohmi, 2001; Silvia, 2005c; Silva, 2006; Avalos et al., 2021), but confusion in the context of art has only recently attracted attention (Silvia, 2010; Silvia and Berg, 2011). Most studies on confusion have focused on facial and other physical expressions (D’Mello and Graesser, 2009) while ignoring the parallel evaluation structure related to confusion and interest (Silvia, 2010). Interest and confusion are closely related to knowledge and emotion (Silvia, 2010), and the influence of expertise on interest and confusion has not been widely studied; however, some researchers have observed differences between experts and novices and indicated that interest and confusion change with expertise. Rich professional knowledge in a related field can make experts notice subtle differences and opinions that novices cannot. Experts often feel that the more they learn, the more complex and mysterious they consider a field. Research also indicates that concepts that confuse novices may interest experts because they are better able to understand them (Silvia, 2005a,b; Silva, 2008; Avalos et al., 2021). For example, experts are more likely to find art interesting (Millis, 2001; Avalos et al., 2021). Regarding confusion, Silvia and Berg (2011) reported that people who knew more about films thought excerpts submitted to local film festivals were more interesting and comprehensible (Silvia, 2006).

### New Media Literacy

The current media environment has certain thresholds and requirements for an ordinary audience to receive art information. The media literacy of an audience has become a precondition for effective integration into the environment. Therefore, in this study, the participation of an audience of digital art was taken as an example in an attempt to add new media literacy to established esthetic models (Avalos et al., 2021) and more comprehensively explore the esthetic model applicable to the new media environment.

The concept of media literacy originated in the 1930s and refers to the ability to access, understand, and create media information in various situations. It has developed rapidly over the past 20 years, and no unified definition is available. Researchers in various countries have constructed various dimensions to describe media literacy on the basis of critical media research theory, new media literacy theory, media theory, and pragmatism theory.

Media literacy can be defined as the expansion of new literacy or cultural literacy (Burnett and Merchant, 2015). Media literacy is closely related to the development of information communication technology. Every leap forward in media communication technology inevitably leads to the deconstruction and reconstruction of individual media literacy (Zhang, 2012). After several paradigm shifts, the research focus on media literacy shifted from describing the audience as simple and passive media consumers to describing users as active media information builders (Peng, 2008). In addition to understanding the meanings and applications of information, skills related to media use, the organization and operation of media, and the motivation to use media, analyzing the specific symbols and symbolic meaning of media information are topics that should be included in media literacy studies (Hobbs, 2000). An individual, whether they are a master or merely a user of such media, should have the enthusiasm and initiative to face the media (Duan et al., 2007). This approach to media literacy also conforms to the new development of an audience entering the field of digital art media.

The birth of the Internet is a milestone in the development of media literacy. The empowering feature of new media is that it enables ordinary users to construct (and coconstruct) media content. As Jenkins (2008) stated, media consumers are no longer at the end of information flows, simply receiving information. Instead, they are actively involved in changing the flow of information through participation and collaboration with other media users.

Therefore, on the basis of current concepts and requirements of media literacy, this study mainly applied the theoretical framework of new media literacy built by Professor Lin Zibin. This is a dynamic theoretical framework for understanding media literacy in the Web 2.0 environment involves using a questionnaire to measure media literacy. This questionnaire has been used by the Ministry of Education of Singapore to test its domestic students. In many tests, the reliability and validity of the theoretical framework and the measurement tool have been confirmed.

**FIGURE 1:**
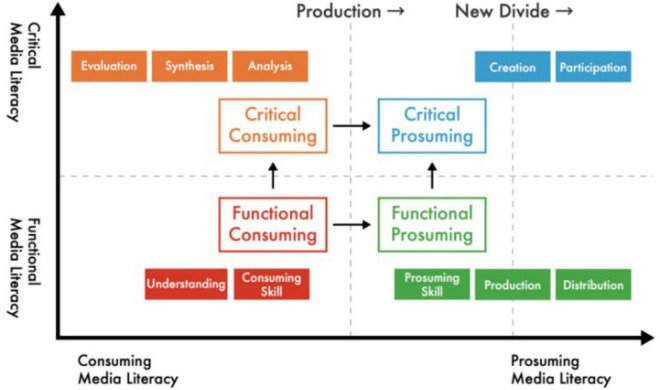
New media literacy model (Lin et al., 2013).

In the theoretical model (Figure 1), new media literacy can be understood as two continua – from consuming literacy to “prosuming consumption” literacy and from functional literacy to critical literacy. These two continuities include four types of literacy: critical consuming literacy, critical prosuming literacy, functional consuming literacy, and functional prosuming literacy.

As shown in the figure, the four quadrants of literacy include ten dimensions of new media literacy. Functional consuming literacy involves the ability to approach the media and understand the information content carried by the media. Critical consuming literacy involves the ability of the audience to analyze, evaluate, and synthesize media content. Critical prosuming literacy involves the ability to participate in media and produce content through media. Functional prosuming literacy involves the ability of users to participate in the production of media content.

Functional media literacy is vital, and it is the starting point of the development of the model because users must be familiar with the technical characteristics of new media tools and new media language to actively use new media technology. The two dimensions, consumption skill and understanding, are the basis of new media literacy. Functional consumption skills refer to a series of technical skills that individuals require to consume media content.

## Construction and Development of the Research Model

**FIGURE 2:**
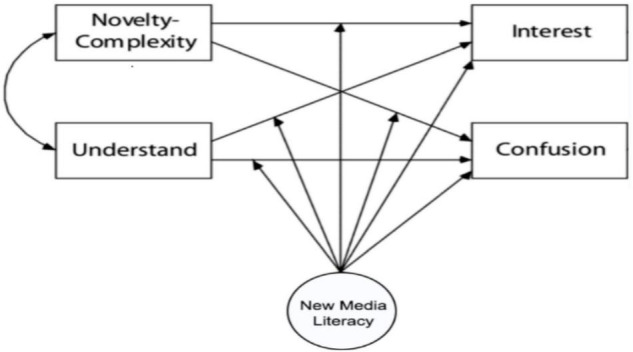
Hypothesis model.

**FIGURE 3:**
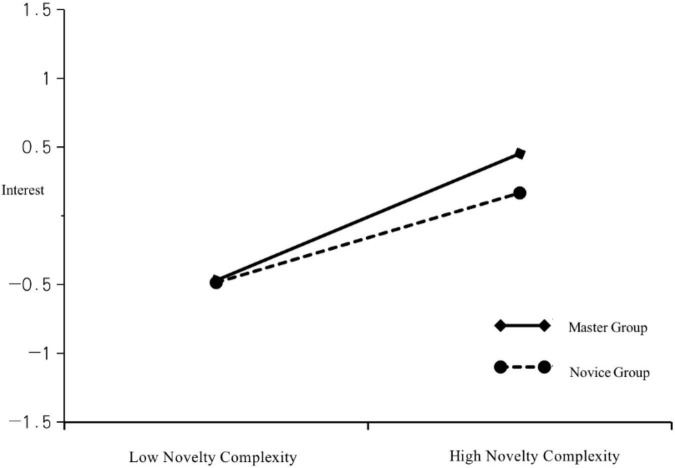
Adjustment chart of group type for interest and novelty–complexity.

To establish a more comprehensive esthetic model that conforms with the esthetic characteristics of modern audiences, this study established an esthetic model with “new media literacy” as the intermediary variable. Figure 2 presents the esthetic model (as shown in Figure 3).

New media literacy requires the audience to have a basic understanding of the operation of new media, be able to search information, and know how to use information technology. This indicator is similar to the access mode in Buckingham et al. (2005), which focuses on the ability to manipulate the hardware and software of access media and collect information. The indicator also includes the access rights of Chen and Wu (Chen et al., 2011), which involve the ability to use different formats or forms of media. With the development of Internet technology, functional consuming skills have become necessary for an audience to enter the field of digital art.

Understanding in new media literacy refers to the ability of the audience to understand the meaning of media content, which overlaps with “comprehensibility” in the original esthetic model, including the ability to capture media content published on different platforms and the ability to interpret the meaning of new forms of expression. In the field of digital art, understanding information content becomes the key to measuring the degree of audience involvement, and the degree of involvement is related to the confusion and interest in this esthetic model. Thus, hypotheses H1 and H2 are proposed:

H1-1: The higher is the audience’s new media literacy, the greater is their interest in new media.

H1-2: The lower is the audience’s new media literacy, the smaller is their interest in new media.

H2-1: When the new media literacy of an audience is high, its confusion is lower.

H2-2: The lower is an audience’s new media literacy, the higher is its confusion.

## Research Methods

The experiment was divided into two stages to test the impact of new media literacy in the esthetic experience model (interest and confusion). In the first stage, we tested the level of new media literacy and divided the participants into groups. In 1998, Singer emphasized the necessity of empirical research in the field of media literacy. Other researchers have agreed and crafted items to measure media literacy for reasonable evaluation (Hobbs, 2000; Primack et al., 2006; Carney, 2008; Tulodziecki, 2012). In view of the unnecessary influence of a pretest on the follow-up experimental results, the post-only control design method was used in the first part of the experiment. Before the experiment, the participants were asked to fill out an online consent form and provide relevant personal information. Because the test sample involved color, the researchers asked for confirmation regarding colorblindness, visual acuity, and corrected visual acuity before the experiment. To better control the sample, the researchers screened it according to personal information, including age, gender, and professional art training (art department and non–art department) so as to reduce the effect of these factors. Most researchers recommend a sample size of 100–200 for factor analysis. In view of this, at this stage of the study, the participants (*N* = 200) were selected according to the aforementioned background. In this study, the media literacy self-assessment scale (MLSS), which has been proven to be reliable and valid, was used to evaluate media literacy. Because of the ongoing pandemic, to improve the efficiency of experimental work, a network survey (Samuel, 2008) was conducted, and the Chinese version of the MLSS was used. The MLSS scale has 13 items. This study mainly discusses the relationship between college students’ media literacy and esthetic model. Therefore, 13 items that are more relevant are screened, mainly including the following items:

m-1-a: I can understand different types of media (such as visual media, audio media) and their principles;
m-1-b: I can understand how to operate and access media;
m-1-c: I can understand the content of media access;
m-2-b: I can use different media technologies to store/back up content.

Data distribution show in Table 1, a total of 385 questionnaires were collected. With the elimination of invalid questionnaires, 233 questionnaires were obtained. Using SPSS 14.0 for data analysis, we observed that in the four new media literacy items of m-1-a, m-1-b, m-1-c, and m-2-b, the lowest score was 4, the highest score was 28, the median score was 20, the average score was 19.64, and the standard deviation was 5.84. In terms of score, the top 25% (Q1 = 16, *n* = 83) and the bottom 25% (Q3 = 24, *n* = 72) of the respondents were assigned to the expert (*n* = 70) and novice (*n* = 70) groups, respectively.

**TABLE 1:** Data distribution.

	Percentiles	Smallest		
1%	4	4	Obs	233
5%	10	4	Sum of Wgt.	233
10%	12	4		
25%	16	5	Mean	19.64378
50%	20	Largest	Std. Dev.	5.843296
75%	24	28		
90%	28	28	Variance	34.14411
95%	28	28	Skewness	−0.2498292
99%	28	28	Kurtosis	2.40836

In the second stage of the experiment, to ensure the credibility of the results, the experimental environment was constrained to exclude factors that might affect the results of the study. The expert (*n* = 70) and novice (*n* = 70) groups were concentrated in a spacious space with a projector (a classroom at a university in Fuzhou). To eliminate the influence of the environment, lighting and other potential interference factors were measured. The participants were asked to observe stimuli in the same classroom at the same period. The groups were asked to log on to the website of *The First Triennial of X Art Museum*. The total observation time was limited to 10 min, and the display time of each image stimulus was approximately 20 s. In the experiment, all the images, videos, and related links observed were connected by a computer (Apple MacBook Pro, 15-inch, mid-2014, resolution of 2880 × 1880) and projected onto a large screen for display (projector: Epson EB-c2040XN; size: 1024 × 768).

The brightness, contrast, and resolution of the screen were adjusted to ensure that the priming stimuli were optimal. After watching the stimuli, the participants responded to the questionnaire items regarding the interest and confusion aspects of the esthetic model (to measure the structure of the research model proposed in this study). The items included interest (interesting–boring, exciting–boring), confusion (familiar–unfamiliar), novelty–complexity (simple–complex, common–special), and comprehensibility (understandable–incomprehensible, easy to understand–difficult to understand). A 7-point Likert-type scale was used for all items, and the score ranged from 1 (*totally disagree*) to 7 (*totally agree*).

## Analysis of Results

### Independent Samples *t*-Test

In questionnaire analysis, the most commonly used difference test is the independent samples *t*-test. The independent samples *t*-test is suitable for assessing the difference of two means, and it is suitable for examining an independent variable and binary discontinuous variable. In this survey, an independent samples *t*-test was used to test for differences in the evaluation of interest, confusion, novelty–complexity, and intelligibility between the expert and novice groups.

Table 2 presents the mean values and standard deviations for the interest, confusion, novelty–complexity, and intelligibility image evaluations of the groups. In the independent samples *t*-test, the p values for interest, confusion, novelty–complexity, and intelligibility were below 0.05, which indicated significant differences. Among these variables, the interest and novelty–complexity scores of the expert group were significantly stronger than those of the novice group; in the evaluation of confusion and intelligibility, the novice group scored significantly higher than did the expert group.

**TABLE 2:** Independent samples *t*-test.

Variable	Group	Number of cases	Average value	Standard deviation	*t* value	*P*-value
Interest	Novice group	70	2.750	1.706	–8.044	0.000
	Master group	70	5.000	1.602		
Confusion	Novice group	70	5.214	1.392	7.807	0.000
	Master group	70	3.343	1.443		
Novelty complexity	Novice group	70	3.550	1.246	–4.519	0.000
	Master group	70	4.486	1.204		
Evaluation of intelligible image	Novice group	70	4.843	1.687	8.038	0.000
	Master group	70	2.843	1.220		

### Correlation Analysis

In statistics, the Pearson product-moment correlation coefficient is also known as the Pearson product-moment correlation coefficient. PPMCC or PCCs) is used to measure the correlation (linear correlation) between two variables X and Y, with a value between -1 and 1.The closer the correlation coefficient is to 1 or -1, the stronger it is, and vice versa. In addition, the correlation coefficient and significance level should be considered comprehensively to evaluate correlations. Only when a correlation coefficient is greater than 0 and the *p* value is < 0.05 can the variables be considered related. Therefore, the researchers used Pearson’s correlation coefficient to verify correlations between variables.

As shown in Table 3, a significant positive correlation was identified between group and interest (*r* = 0.565, *p* < 0.01), a significant negative correlation was identified between group and confusion (*r* = −0.553, *p* < 0.01), a significant positive correlation was identified between group and novelty–complexity (*r* = 0.359, *p* < 0.01), and a significant negative correlation was identified between group and intelligibility evaluation (*r* = −0.565, *p* < 0.01). A significant negative correlation was identified between interest and confusion (*r* = −0.763, *p* < 0.01), a significant positive correlation was identified between interest and novelty–complexity (*r* = 0.627, *p* < 0.01), and a significant negative correlation was identified between interest and intelligibility evaluation (*r* = −0.760, *p* < 0.01). Confusion was negatively correlated with novelty–complexity (*r* = −0.477, *p* < 0.01) and positively correlated with the evaluation of intelligibility (*r* = 0.612, *p* < 0.01). A significant negative correlation was identified between novelty–complexity and resolvable image evaluation (*r* = −0.465, *p* < 0.01).

**TABLE 3:** Correlation analysis.

	Group	Interest	Confusion	Novelty complexity	Evaluation of intelligible image
Group	1				
Interest	0.565**	1			
Confusion	−0.553**	−0.763**	1		
Novelty complexity	0.359**	0.627**	−0.477**	1	
Evaluation of intelligible image	−0.565**	−0.760**	0.612**	−0.465**	1

*p < 0.05, **p < 0.01.

### Regression Analysis

Regression analysis is a statistical method used to determine the interdependent relationship between two or more variables. Regression analysis was used to test whether novelty–complexity and the evaluation of the intelligibility of images had significant effects on interest and confusion.

#### Evaluation of Novelty–Complexity and Intelligibility and Regression Analysis of Interest

As shown in Tables 4–6, the explanation rate of novelty complexity to interest was 66.9% in the evaluation of intelligible images, and a significant linear relationship existed between novelty–complexity and interest in the evaluation of image intelligibility (*F* = 141.331, *p* < 0.001). Among the variables, novelty–complexity had a significant positive impact on interest (β = 0.349, *p* < 0.001); the evaluation of intelligibility had a significant negative impact on interest (β = −0.598, *p* < 0.001).

**TABLE 4:** Model summary.

Model	R	R square	Adjusted R square	Errors in standard estimates
1	0.82l^a^	0.674	0.669	1.15006

*^a^Predictive variables (constant): evaluation of intelligible images, novelty complexity.*

**TABLE 5:** ANOVA*^a^*.

Model		Quadratic sum	Degree of freedom	Mean square	F	Significance
	Regression	373.861	2	186.930	141.331	0.000^b^
1	Residual error	181.202	137	1.323		
	Total	555.063	139			

*^a^Dependent variable: interest.*

*^b^Predictive variables (constant): evaluation of intelligible images, novelty complexity.*

**TABLE 6:** Coefficients*^a^*.

Mode		Non-standardized coefficient		Standardized coefficient		Significance
			
		B	Standard error	Beta	*t*	
	(Constant)	4.313	0.506		8.529	0.000
1	Novelty complexity	0.534	0.084	0.349	6.338	0.000
	Evaluation of intelligible image	−0.672	0.062	−0.598	−10.844	0.000

*^a^Dependent variable: interest.*

#### Regression Analysis of Confusion Based on the Evaluation of Novelty–Complexity and Resolvable Image

As shown in Tables 7–9, the explanation rate of novelty complexity to confusion was 41.4% in the evaluation of intelligible images, and a significant linear relationship existed between novelty–complexity and confusion in the evaluation of intelligibility (*F* = 50.070, *p* < 0.001). Among the variables, novelty–complexity had a significant negative effect on confusion (β = −0.246, *p* < 0.01), and the evaluation of intelligible images had a significant positive effect on confusion (β = 0.498, *p* < 0.001).

**TABLE 7:** Model summary.

Model	R	R square	Adjusted R square	Errors in standard estimates
1	0.650*^a^*	0.422	0.414	1.29898

*^a^Predictive Variables (constant): Evaluation of intelligible images and novelty complexity.*

**TABLE 8:** ANOVA*^a^*.

Model		Quadratic sum	Degree of freedom	Mean square	*F*	Significance
1	Regression	168.970	2	84.485	50.070	0.000*^b^*
	Residual error	231.165	137	1.687		
	Total	400.136	139			

*^a^Dependent variable: confusion.*

*^b^Predictive variables (constant): Evaluation of intelligible images and novelty complexity.*

**TABLE 9:** Coefficient*^a^*.

Model		Non-standardized coefficient		Standardization coefficient		Significance
			
		B	Standard error	Beta	*t*	
1	(Constant)	3.733	0.571		6.537	0.000
	Novelty complexity	−0.319	0.095	−0.246	−3.353	0.001
	Evaluation of Intelligible image	0.475	0.070	0.498	6.791	0.000

*^a^Dependent variable: confusion.*

#### Group Moderation of Novelty–Complexity, Intelligible Image Evaluation, and Interest

As Table 10 indicates, the increase of novelty complexity and interaction terms of group, the evaluation degree of cleavable images and the interaction term R^2^ of the group are significantly improved (△R^2^ = 0.014, *p* < 0.05). Novelty complexity and grouped interaction items had significant positive effects on interest (β = 0.135, *p* < 0.05). Evaluation of the intelligibility of images and the interaction of the group (expert vs. novice) had no significant effect on interest (β = 0.058, *p* > 0.05); therefore, group type had a positive moderating effect on novelty–complexity and interest but had no moderating effect on the relationship between intelligibility and interest. To further test the moderating effect of the group on novelty–complexity and interest, we used a moderating effect diagram and Process (version 3.3) for simple slopes analysis with 5000 bootstraps.

**TABLE 10:** Moderating effects of novelty–complexity, intelligible image evaluation, and interest.

Variable	Model I		Model II		Model III	
			
	β	*t*	β	*t*	β	*t*
Novelty complexity	0.349	6.338***	0.331	6.069***	0.326	6.065***
Evaluation of intelligible image	−0.598	−10.844***	−0.520	−8.449***	−0.527	−8.321***
Group			0.152	2.607**	0.151	2.570*
Novelty complexity* group					0.135	2.513*
Evaluation of intelligible image* group					0.058	0.916
R^2^	0.674		0.689		0.703	
adj/R^2^	0.669		0.682		0.692	
△R^2^	0.674		0.016*		0.014*	
*F*	141.331***		100.473***		63.472***	

*p < 0.05, **p < 0.01, ***p < 0.001.

Table 11 contains the results of the simple slope analysis. In the novice group, novelty–complexity had a significant positive effect on interest. The simple slope estimation was 0.191 (*p* < 0.05), and the confidence interval did not include 0 (0.038, 0.344); after adjustment, the estimate was 0.461 (*p* < 0.001), and the confidence interval did not include 0 (0.313, 0.608; Figure 3). Novelty–complexity had a negligible positive effect on interest in the novice group, and the slope was gentle. In the expert group, novelty–complexity had a greater positive effect on interest, and the slope was steep. Therefore, grouping had a significant positive regulatory effect on the relationship between novelty–complexity and interest.

**TABLE 11:** Simple slopes analysis.

Group	Effect	se	*t*	*P*	LLCI	ULCI
Novice group	0.191	0.077	2.470	0.015	0.038	0.344
Master group	0.461	0.075	6.170	0.000	0.313	0.608

#### Adjustments of Group in the Evaluation of Novelty–Complexity, Intelligibility, and Confusion

As is shown in Table 12, the R^2^ value was significantly improved in increasing novelty complexity and group interaction items, evaluation degree of resolvable image and grouping interaction items (△R^2^ = 0.034, *p* < 0.05). Novelty–complexity and group interaction had significant negative effects on confusion (β = −0.162, *p* < 0.05). The degree of image intelligibility and the interaction item of grouping had a significant effect on confusion (β = 0.092, *p* > 0.05). Thus, grouping had a negative moderating effect on novelty–complexity and confusion but had no moderating effect on intelligibility and confusion. To further test the moderating effect of grouping on novelty–complexity and confusion, a simple slopes analysis was performed using a moderating effect diagram and Process (3.3) with 5000 bootstraps.

**TABLE 12:** Adjustments for group type in the evaluation of novelty–complexity, intelligibility, and confusion.

Variable	Model I		Model II		Model III	
			
	β	*t*	β	*t*	β	*t*
Novelty complexily	−0.246	−3.353**	−0.212	−2.988**	−0.213	−3.076**
Evaluation of intelligible image	0.498	6.791***	0.359	4.470***	0.414	5.076***
Group			−0.275	−3.612***	−0.243	−3.222**
Novelty complexity* group					−0.162	−2.338*
Evaluation of intelligible image* group					0.092	1.128
*R* ^2^	0.422		0.473		0.507	
adj/R^2^	0.414		0.461		0.489	
△R^2^	0.422		0.051***		0.034*	
*F*	50.070***		40.665***		27.573***	

*p < 0.05, **p < 0.01, ***p < 0.001.

**FIGURE 4:**
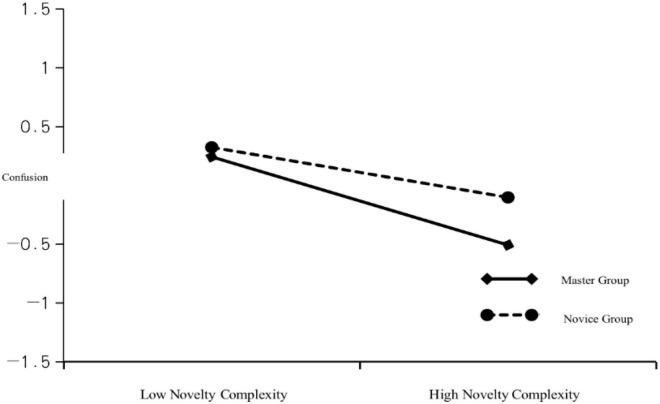
Adjustment chart of grouping for novelty–complexity and confusion.

Table 13 presents the results of the simple slopes analysis. In the novice group, novelty–complexity had no significant negative effect on confusion. The simple slopes estimation was −0.051 (p > 0.05), and the confidence interval did not include 0 (−0.248, 0.146). In the expert group, novelty–complexity had a significant negative effect on confusion; the simple slope was −0.375 (*p* < 0.001), and the confidence interval did not include 0 (−0.565, −0.184). As Figure 4 suggests, novelty–complexity had a negligible negative effect on confusion in the novice group (the slope was gentle). In the expert group, novelty–complexity had a greater negative effect on confusion (the slope was steep). Therefore, grouping had a significant negative moderating effect on the relationship between novelty–complexity and confusion. Considering that this experiment mainly discusses and analyzes college students’ esthetic appreciation and media literacy in the digital field, it is highly subjective, and it is normal for some insignificant phenomena to occur in relevant experiments. In this paper, a good demonstration of the role of research.

**TABLE 13:** Simple slope analysis.

Group	Effect	se	*t*	*P*	LLCI	ULCI
Novice group	−0.051	0.100	−0.513	0.609	−0.248	0.146
Master group	−0.375	0.096	−3.894	0.000	−0.565	−0.184

## Conclusions and Prospects

The research group recruited a group of college students as the main research object and, through combing through esthetic model theory and the theoretical framework of the esthetic model, discussed the theoretical and practical significance of new media literacy as a moderating variable. The aim was to improve the developing esthetic model so that it can be more suitable for application with audiences in the new media era.

The statistical results indicate that the hypotheses and measurement indicators reasonably measured and explained new media literacy as a moderator in the esthetic model and had good reliability and validity. The results demonstrate the research hypothesis that the higher is an audience’s new media literacy, the greater are their esthetic interest and confusion. By contrast, the lower is an audience’s new media literacy, the lower are their esthetic interest and confusion. This indicates that audience cognition in the digital art field requires both cognitive ability and new media literacy. This also confirms the importance of media literacy level, It can be imagined that with the increasing popularity of digitalization, media literacy plays an important role for college students, especially in the fields of information cognition, recognition, screening and aesthetics.

The art field will continue to change with culture and technology. An invariable esthetic model would limit the esthetic expression of audiences and creators. The proposal of new media literacy in an esthetic model is closely related to ongoing new media developments. With the diverse expression of art in the digital field, new media literacy has become essential for audiences. Improving esthetic ability and new media literacy through improving self-literacy will become key activities of contemporary audiences. Taking college students who use new media more as an example, the paper aims to summarize the rules and gradually improve the developing esthetic model so as to make it more suitable for the esthetic application of readers and listeners in the new media era. Based on the study of college students, further exploration of the esthetic experience model of readers in the digital environment can provide theoretical support and transcendental research for the later education and teaching reform of new media literacy in universities. In addition, the findings indicate that with different discipline backgrounds, new media literacy differed; these findings open new ideas for further research. For example, research can provide the foundation for the development of an esthetic model for a more diverse disciplinary background and further expand the cognition and development aspects of the aforementioned model.

The deficiency of this study is that although it proved that new media literacy influences esthetic cognition, only an audience of college students was studied. But under the current global digital background, the discussion of college students alone is not enough to represent the vast number of new media users. The original intention of this study is to provide a reference for the whole people to participate in digital art by new discussion of esthetic model combined with new media literacy. The researchers anticipate that the research sample can be expanded to make the results more convincing and representative. For example, expanding the range of majors that students can study in the digital field, including students from different countries.

## Data Availability Statement

The original contributions presented in this study are included in the article/supplementary material, further inquiries can be directed to the corresponding author.

## Author Contributions

CW and RX: conceptualization and investigation. YH: methodology. RX: software, resources, data curation, writing—original draft preparation, visualization, and funding acquisition. CW, RX, and YH: validation. CW: formal analysis, writing—review and editing, and project administration. CW and YH: supervision. All authors have read and agreed to the published version of the manuscript.

## Conflict of Interest

The authors declare that the research was conducted in the absence of any commercial or financial relationships that could be construed as a potential conflict of interest.

## Publisher’s Note

All claims expressed in this article are solely those of the authors and do not necessarily represent those of their affiliated organizations, or those of the publisher, the editors and the reviewers. Any product that may be evaluated in this article, or claim that may be made by its manufacturer, is not guaranteed or endorsed by the publisher.
